# Synthesis of Sub 3 nm-Sized Uniform Magnetite Nanoparticles Using Reverse Micelle Method for Biomedical Application

**DOI:** 10.3390/ma12233850

**Published:** 2019-11-22

**Authors:** Euiyoung Jung, Sung-Won Kim, Ahyoung Cho, Yu-Jin Kim, Gun-Jae Jeong, Jinheung Kim, Suk Ho Bhang, Taekyung Yu

**Affiliations:** 1 Department of Chemical Engineering, Kyung Hee University, Yongin 17104, Korea; jey9207@khu.ac.kr (E.J.); cayoung15@khu.ac.kr (A.C.); 2 Department of Chemistry and Nano Science, Ewha Womans University, Seoul 03760, Korea; 3 School of Chemical Engineering, Sungkyunkwan University, Suwon 16419, Korea; tjdnjdl90@skku.edu (S.-W.K.); yujinkim1003@gmail.com (Y.-J.K.); 4 Division of Vascular Surgery, Samsung Medical Center, Sungkyunkwan University School of Medicine, Seoul 06351, Korea; jgj814@skku.edu

**Keywords:** iron oxide, magnetite, nanoparticles, reverse micelle, sub-3 nm

## Abstract

We report a synthetic method for small and uniform Fe_3_O_4_ (magnetite) nanoparticles under mild conditions. Spherical sub-3 nm-sized magnetite nanoparticles were prepared via reverse micelles composed of oleylamine, F127, xylene, and water for the reaction of iron(III) stearate with hydrazine at a reaction temperature of 90 °C in air atmosphere. These synthesized magnetite nanoparticles exhibited good size uniformity. By controlling experimental conditions, we could easily control both size and size uniformity of these magnetite nanoparticles. We further investigated whether Fe_3_O_4_ could be used in biomedical applications. Cytotoxicity of Fe_3_O_4_ was evaluated with human adipose-derived stem cells (hADSCs). Our results showed that the number of hADSCs did not significantly decrease when these cells were treated with Fe_3_O_4_ nanoparticles at a concentration of up to 9 μg/mL. Apoptotic activity and cell proliferation of hADSCs treated with Fe_3_O_4_ nanoparticles were similar to those of hADSCs without any treatment. This novel method could be used for synthesizing uniform and biocompatible Fe_3_O_4_ nanoparticles with further biomedical applications.

## 1. Introduction

Small nanoparticles with sizes of less than 5 nm have received enormous attention from both scientific and industrial areas due to their specific size-dependent magnetic, optical, electrical, and catalytic characteristics that their bulk counterparts do not possess [1,2,3,4]. For example, magnetic iron (Fe) oxide nanoparticles exhibit superparamagnetism by the Neel and Brown relaxation effect induced by thermal fluctuation when their sizes are decreased [5,6,7,8]. These ultra-small iron oxide nanoparticles with sizes of less than 5 nm have been used as highly sensitive magnetic resonance imaging (MRI) contrast agents for the imaging of blood vessels with small sizes [7,8,9,10,11,12]. Size uniformity has also received attention because the superparamagnetic properties of small iron oxide nanoparticles strongly depend on their sizes [9]. Although there have been many methods for synthesizing small iron oxide nanoparticles, including hydrothermal, solvothermal, and thermal decomposition methods, these methods require harsh reaction conditions involving the use of toxic organic solvents, high reaction temperatures, or high pressure [13,14,15]. Therefore, it remains a great challenge to develop a simple and reliable route to synthesize small and uniform iron oxide nanoparticles under mild reaction conditions.

To synthesize nanoparticles in mild reaction conditions including a low temperature under 100 °C and air atmosphere, reverse micelles that are surfactant-immobilized water-in-oil emulsions have been typically used as nanoreactors for the synthesis of metal nanoparticles [16,17,18,19,20,21,22]. However, metal oxide nanoparticles synthesized using these processes often suffer from poor crystallinity due to low reaction temperatures. Consequently, heat treatment post-synthesis is necessary to obtain highly crystalline nanoparticles. Recently, Hyeon and co-workers [23] have reported the synthesis of monodisperse magnetite nanoparticles using the reverse micelle method. However, the sizes of synthesized nanoparticles were larger than 5 nm. Some other groups have tried to synthesize very small iron oxide nanoparticles with uniform sizes, but the synthetic processes are still complicated and difficult to control [24,25,26]. Therefore, a new general method for the synthesis of small and uniform metal oxide nanoparticles under mild synthetic conditions needs to be developed for biological and environmental applications. We herein report the synthesis of sub-3 nm-sized uniform magnetite nanoparticles from a reaction between Fe (III)-stearate and a reactant in reverse micelles composed of oleylamine, F127, xylene, and water at reaction temperature of 90 °C in air atmosphere. Despite such a low reaction temperature, these synthesized 3 nm-sized spherical magnetite nanoparticles exhibited good size uniformity and a highly crystalline nature. We also investigated the role of oleylamine and F127 in the synthesis. We were able to control the size of these nanoparticles by changing the carboxylate ion of the Fe(III) precursor.

In the present study, we also tested whether manufactured Fe_3_O_4_ nanoparticles could be potentially used in biomedical applications. Human adipose-derived stem cells (hADSCs) have been widely used for both research and clinical trials. Thus, these cells were used with our newly synthesized Fe_3_O_4_ nanoparticles. The number of hADSCs treated with Fe_3_O_4_ nanoparticles at a concentration of up to 30 µg/mL is similar to that of hADSCs without treatment. Apoptotic activity and cell proliferation behavior of hADSCs were determined at both gene and protein levels after the cells were exposed to Fe_3_O_4_ nanoparticles. Apoptotic activity and cell proliferation of hADSCs treated with Fe_3_O_4_ nanoparticles at a concentration of up to 30 µg/mL were similar to those of hADSCs without treatment. Taken together, our results demonstrate that the novel method discovered in this research can be utilized for synthesizing uniform and biocompatible Fe_3_O_4_ nanoparticles with further biomedical applications.

## 2. Materials and Methods

### 2.1. Materials

Iron(III) stearate (Fe(C_18_H_35_O_2_)_3_), iron(III) acetylacetonate (Fe(C_5_H_7_O_2_)_3_), iron(III) chloride hexahydrate (FeCl_3_·6H_2_O), oleylamine, oleic acid, hydrazine monohydrate (NH_2_NH_2_·H_2_O), hydrogen peroxide (H_2_O_2_), F127, xylene, and PEG (MW 4000) were purchased from Sigma–Aldrich (Louis, MO, USA) and used without further purification. Oleic acid sodium salt was purchased from TCI (Tokyo Chemical Industry, Tokyo, Japan).

### 2.2. Synthesis of Magnetite Nanoparticles

To obtain sub-3 nm-sized magnetite nanoparticles, 1 g of F127 was dissolved in 5 mL of water. A mixture solution was prepared by mixing 0.5 mmol of Fe(III) stearate (0.453 g), 1 mmol of olyelamine, and 15 mL of xylene. It was then added to F127 aqueous solution under magnetic stirring. For stabilization, the reacting solution was kept at room temperature for 12 h. Then, the reverse micelle solution was gradually heated to 90 °C and 0.2 mL of hydrazine (11 wt % water solution) was rapidly injected into the reverse micelle solution. The resulting solution was heated at the same temperature for 3 h. An aliquot of the solution was dispersed in chloroform and washed with acetone.

### 2.3. Synthesis of Fe (III)-Oleate

To prepare Fe(III) oleate complex, 40 mmol of FeCl_3_·6H_2_O and 36.54 g of oleic acid sodium salt (120 mmol) were added to a mixture solution composed of ethanol (80 mL), distilled water (60 mL), and n-hexane (140 mL). The resulting solution was heated to 70 °C and maintained at this temperature for 4 h. The solution was then transferred to a separatory funnel. The upper organic layer containing the Fe(III) oleate complex was then separated and washed several times with DI water. The resulting waxy black Fe(III) oleate complex was obtained after evaporation of n-hexane.

### 2.4. Ligand Modification

Magnetite nanoparticles were dried overnight in the oven at 70 °C. Dried magnetite nanoparticles were stirred in a PEG4000 solution in distilled water at 50 °C until dispersion occurred. Then, magnetite nanoparticles were diluted in the serum-free cell culture medium for further cytotoxicity tests.

### 2.5. Characterization

Transmission electron microscope (TEM) images, high-resolution TEM (HRTEM) images, and electron diffraction (ED) patterns were captured using a JEM-2100F microscope (JEOL, Tokyo, Japan) operated at 200 kV. 

### 2.6. Cell Culture

The hADSCs used in this study were purchased from Lonza (Bazel, Switzerland). The hADSCs were seeded in a culture medium that includes 10% (*v/v*) fetal bovine serum (Gibco BRL) and 1% (*v/v*) penicillin/streptomycin (Gibco BRL) in Dulbecco’s modified Eagle’s medium (DMEM, Gibco BRL, Gaithersburg, DC, USA) at 37 °C with 5% CO_2_ saturation. hADSCs within passage 6 were used and the culture medium was exchanged once every two days in this study. Cell counting was performed using a cell counting kit-8 (CCK-8, Dojindo Molecular Technologies, Inc., Kumamoto, Japan). The amount of formazan dye is proportional to the number of cells by means of the reduction of formazan dye attributed to intracellular dehydrogenase activity in living cells. Absorbance was measured at 450 nm by a plate reader (Infinite F50, Tecan, Männedorf, Switzerland). Further apoptotic activity of the hADSCs was evaluated with terminal deoxynucleotidyl transferase dUTP nick end labeling (TUNEL, Apop Tag®, Millipore Corp., Billerica, MA, USA) kit.

### 2.7. Reverse Transcription Polymerase Chain Reaction (RT-PCR)

A total of 1 mL of trizol reagent (Life Technologies, Inc., Carlsbad, CA, USA) was treated to lyse hADSCs cultured in a 6-well culture plate. The resultant RNA was extracted by using isopropanol. A 75% ethanol solution in water was added to wash the RNA pellets and the washed RNA pellets were air-dried. Then, 0.1% (*v*/*v*) diethyl pyrocarbonate-treated water was used to dry the pellets. Reverse transcription was performed using 10 μL of 2× Easy Taq SuperMix (TransGen Biotechnology, Beijing, China), 0.5 μL of each primer, 0.5 μL of cDNA, and 8.5 μL of sterile pure H_2_O. Then, PCR amplification was conducted with the resultant solution to amplify synthesized complementary deoxyribonucleic acid. PCR consisted of 35 cycles of denaturing (94 °C, 30 s), annealing (58 °C, 45 s), and extension (72 °C, 45 s), with a final extension step at 72 °C for 10 min. For visualization, PCR products were transported in 2% (*w/v*) gel electrophoresis and highlighted with ethidium bromide. PCR product bands were detected by a gel documentation system (WGD-30, Daihan Scientific, Wonju-Si, Korea) with adequate exposure. β-actin, the house keeping gene, was served as a control. All primers used for RT-PCR assay in this study are shown in Table 1.

### 2.8. Quantitative Real-Time Polymerase Chain Reaction (qRT-PCR)

qRT-PCR analysis was performed to quantify the relative gene expression levels of representative pro-apoptotic and anti-apoptotic genes, *BCL-2* and *BAX* (n = 5). The total ribonucleic acid (RNA) was extracted from lysed samples using 200 μL chloroform after lysing hADSCs with 1 mL trizol reagent. The resultant samples were centrifuged at 12,000 rpm for 10 min at 4 °C. The precipitated RNA pellet was washed with 75% (*v*/*v*) ethanol in water and dried. RNase-free water was used to dissolve the washed pellet right after the drying process. A 10 μL iQ™ SYBR Green Supermix kit (Bio-Rad, Hercules, CA, USA) was added to 10 μL of cDNA, primers and RNase-free water and the resultant mixed solution was analyzed by MyiQ™ single color real-time PCR detection system (Bio-Rad) for qRT-PCR. All primers used for qRT-PCR analysis in this study are written in Table 1.

### 2.9. Western Blot Analysis

All samples were lysed in ice-cold lysis buffer (15 mM Tris-HCl, pH 8.0, 0.25 M sucrose, 15 mM NaCl, 1.5 mM MgCl_2_, ethylenediaminetetraacetic acid, 2.5 mM ethylenediaminetetraacetic acid, 1 mM ethylene glycol tetraacetic acid, 2 mM NaPPi, 1 mM dithiothreitol, 2 mM NaPPi, 1 mg/mL of pepstatin A, 2.5 mg/mL of aprotinin, 0.5 mM phenylmethyl sulfonyl fluoride, 5 mg/mL of leupeptin, 0.5 mM phenylmethyl sulfonyl fluoride, 0.125 mM Na_3_VO_4_, 25 mM NaF, and 10 mM lactacystin). Bicinchoninic acid protein assay (Pierce Biotechnology, Rockford, IL, USA) was performed to decide the concentration of the protein in each sample. Then, Western blot analysis was conducted with 10% sodium dodecyl sulfate-polyacrylamide gel electrophoresis. Transported proteins in the gels were transferred onto an Immobilon-P membrane (Millipore Corp.). Thereafter, transferred proteins were immersed in 5% skim milk in tris-buffered saline and 0.1% Tween® 20 detergent (TBST) buffer for 1 h at room temperature for blocking, and probed with first antibodies against caspase-3, proliferating cell nuclear antigen (PCNA), and GAPDH overnight at a temperature of 4 °C. After replenishing the first antibodies on the blots with TBST buffer, the blots were incubated with a horseradish peroxidase-conjugated secondary antibody (Santa Cruz Biotechnology, Santa Cruz, CA, USA) for 1 h at room temperature. Thereafter, blots were developed using an enhanced chemiluminescence detection system (Amersham Bioscience, Piscataway, NJ, USA) and luminescence was recorded on the X-ray film (Fuji super RX, Fujifilm Medical Systems, Tokyo, Japan). Bands were imaged and quantified (n = 5 different samples per group) by using an Imaging Densitometer (Bio-Rad, Hercules, CA, USA).

### 2.10. Statistical Analysis

Quantitative data are expressed as mean ± standard deviation. Statistical analysis was performed using an analysis of variance (ANOVA) test followed by a Bonferroni test. A *p* value of less than 0.05 was considered statistically significant.

## 3. Results and Discussion

### 3.1. Characterizaion of the Magnetite Nanoparticles

**Figure 1:**
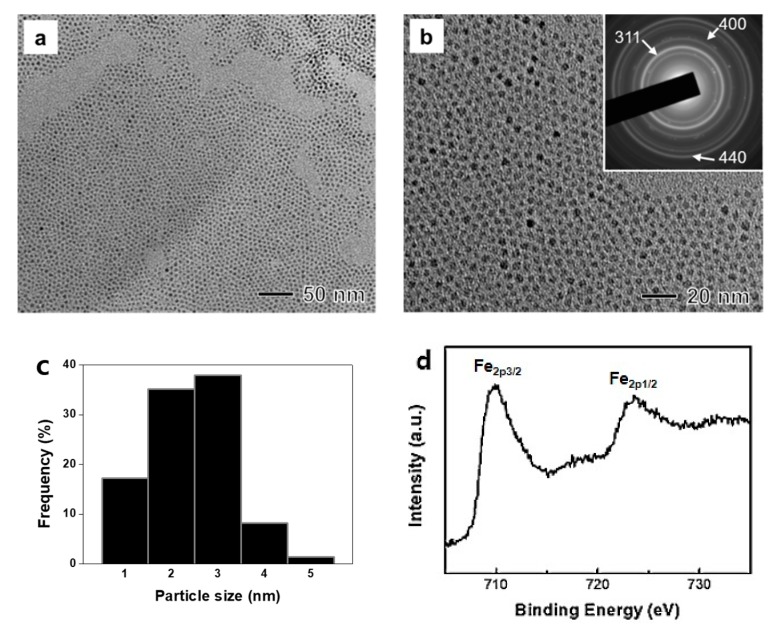
(**a**,**b**) TEM images, ED patterns (inset of Figure 1b), (**c**) size distribution, and (**d**) XPS spectrum of magnetite nanoparticles synthesized by reacting Fe (III)-stearate with hydrazine in reverse micelles composed of oleylamine, F127, xylene, and water at 90 °C for 3 h.

Sub-3 nm-sized magnetite nanoparticles were synthesized by reacting hydrazine with Fe(III) stearate immobilized in reverse micelles composed of oleylamine, F127, xylene, and water. To produce magnetite nanoparticles, hydrazine was used as a reactant with Fe ions [23]. After the addition of hydrazine, the color of the resulting solution quickly changed from red–brown to black. A typical TEM image of the synthesized nanoparticles showed the formation of a spherical shape with an average size of 2.8 nm (Figure 1a,c). As shown in Figure 1b, the synthesized nanoparticles showed very good size uniformity, revealing the formation of monodisperse nanoparticles. ED patterns of these synthesized nanoparticles showed a presence of (311), (400), and (440) planes of face-centered cubic Fe_3_O_4_ (magnetite), demonstrating the formation of magnetite nanoparticles (inset of Figure 1b). We additionally measured XPS spectrum of Fe 2p core level to distinguish Fe_3_O_4_ and γ-Fe_2_O_3_ because they have similar ED patterns. In Figure 1c, 2p_3/2_ and 2p_1/2_ of Fe showed two kinds of sharp peaks between 705 and 735 eV without a satellite peak around 719.2 eV, indicating the crystal structure of Fe_3_O_4_ [27]. Before applying further bio-experiments, ligand modification of Fe_3_O_4_ nanoparticles was conducted by using PEG in order to induce hydrophilic conditions. The average size of the Fe_3_O_4_ nanoparticles by dynamic light scattering was 347.3 nm due to the presence of F127, oleyamine, and PEG on the surface of the Fe_3_O_4_ nanoparticles. The zeta potential value was −2 mV owing to the negative charge functional group of PEG.

### 3.2. Study on the Role of Stabilizer

**Figure 2:**
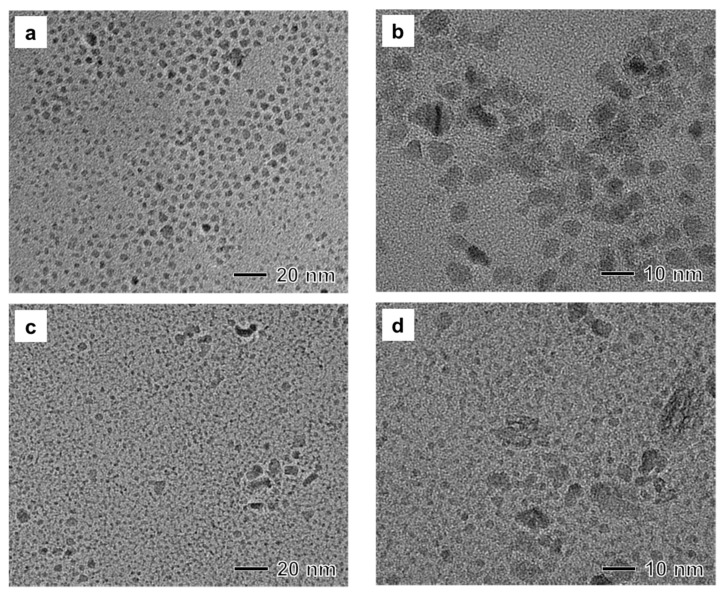
(**a**,**b**) TEM images of a sample produced under the same reaction conditions as those in Figure 1 except for the absence of oleylamine. (**c**,**d**) TEM images of a sample produced under the same reaction conditions as those in Figure 1 except for the absence of F127.

The synthesis involved two stabilizers, oleylamine and F127. To understand the role of these stabilizers for the synthesis of nanoparticles in reverse micelles, we performed control experiments in the absence of oleylamine or F127 while keeping other reaction conditions the same as those in the synthesis of magnetic nanoparticles shown in Figure 1. When the synthesis was conducted in the absence of oleylamine, the sizes of synthesized nanoparticles increased from 1 nm to 10 nm (Figure 2a). In HRTEM imaging, branched polycrystalline nanoparticles were also observed (Figure 2b), revealing that oleylamine could act as a major surfactant for the synthesis of monodisperse nanoparticles. On the other hand, when the synthesis was conducted without F127, we could not prepare a stable reverse micelle solution by mixing xylene and water. After the reaction, TEM images of the product showed the formation of small worm-like nanoparticles and large irregular-shaped nanoparticles. Their crystallinity was very low (Figure 2c,d). Thus, F127 in the aqueous-phase could immobilize reverse micelles in xylene solvent during the present synthesis.

### 3.3. Influence of Precursor

**Figure 3:**
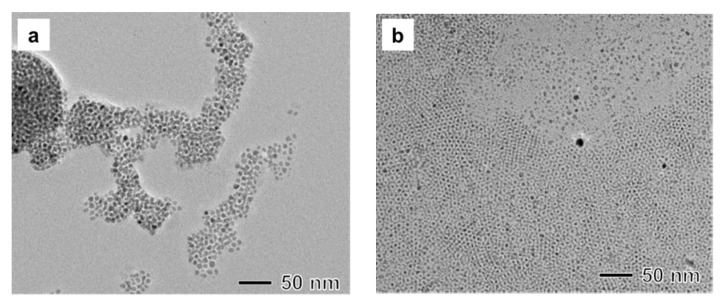
(**a**) TEM image of a sample manufactured under the same reaction conditions as those in Figure 1 except that Fe(III) oleate was used for a precursor instead of Fe(III) stearate. (**b**) TEM image of a sample manufactured under the same reaction conditions as those in Figure 1 except that Fe(III) acetylacetonate and oleic acid were used instead of Fe(III) stearate.

In our study, we used Fe(III) stearate as an Fe precursor. A small amount of stearic acid might be present in the reacting solution. It is well known that carboxylic acid can act as a surfactant for the synthesis of metal oxide nanoparticles [7]. Additionally, previous research has shown that stearic acid can lead to the formation of small nanoparticles compared to oleic acid [28]. When Fe(III) oleate instead of Fe(III) stearate is applied, the size of the synthesized nanoparticle increased to 7 nm. This result demonstrates that the size of magnetite nanoparticles can be controlled by varying carboxylate ions in the Fe(III) precursor (Figure 3a). In addition to the capping and stabilizing effect of carboxylate ions, we also investigated the size control effect by manipulating the reaction rate between an Fe(III) precursor and hydrazine. Typically, when the surfactant forms a complex with a metal precursor, reactivity of the precursor decreases [29]. When the synthesis was conducted using a mixture of Fe(III) acetylacetonate and oleic acid instead of Fe(III) oleate, small magnetite nanoparticles with sizes of less than 2 nm were synthesized (Figure 3b). A fast reaction rate of Fe(III) acetylacetonate with hydrazine would generate a large number of nuclei due to the higher supersaturation in the nucleation stage, leading to the formation of small nanoparticles compared with Fe(III) oleate or stearate.

### 3.4. Toxicity Study of the Synthesized Magnetite Nanoparticles

**Figure 4:**
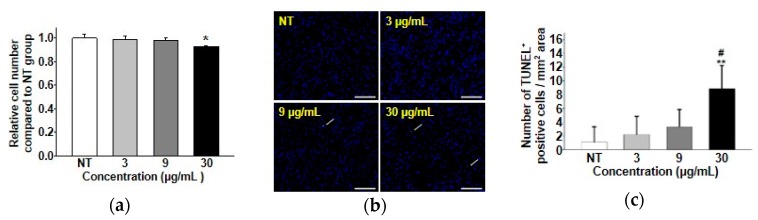
Cytotoxicity of Fe_3_O_4_ nanoparticles to hADSCs depending on concentration used for treatment. (**a**) Cell viability of hADSCs treated with or without Fe_3_O_4_ nanoparticles (* *p* < 0.05 versus all other groups). (**b**) Apoptosis of hADSCs determined by TUNEL assay that stains apoptotic cells (white arrows, green). Blue (DAPI) indicates nuclei of hADSCs. (Scale bar indicates 100 μm) (**c**) Quantification of TUNEL-positive hADSCs per unit area (** *p* < 0.01 versus NT group, # *p* < 0.05 versus 9 μg/mL group).

**Figure 5:**
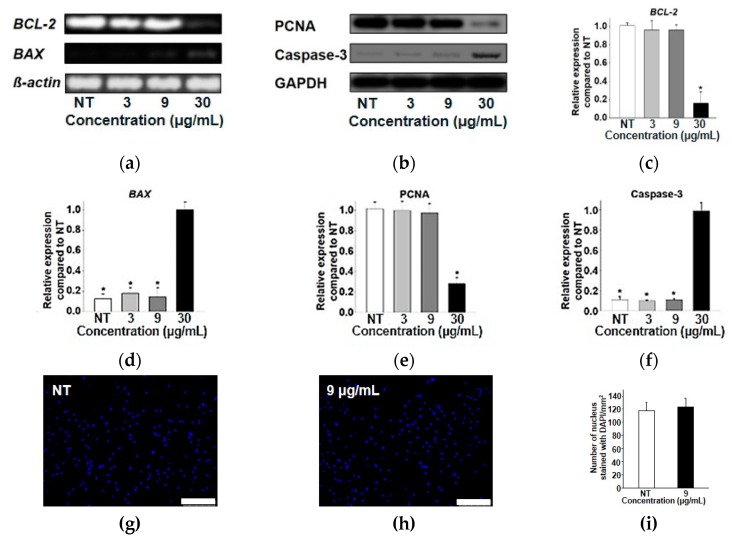
Apoptotic activity in hADSCs treated with Fe_3_O_4_ nanoparticles at different concentrations. (**a**) One of the representative pro-apoptotic (*BAX*) and anti-apoptotic (*BCL-2*) gene expressions in hADSCs treated with or without Fe_3_O_4_ nanoparticles as evaluated by RT-PCR. (**b**) Cell proliferation (PCNA) and pro-apoptotic protein expression in hADSCs treated with or without Fe_3_O_4_ nanoparticles as performed by Western blot analysis. Quantification of (**c**) *BCL-2* (pro-apoptotic) gene expression (* *p* < 0.05 versus all other groups) and (**d**) *BAX* (anti-apoptotic) gene expression (* *p* < 0.05 versus 30 μg/mL groups) using qRT-PCR in hADSCs treated with or without Fe_3_O_4_ nanoparticles. Quantification of (**e**) cell proliferation (PCNA) protein expression (* *p* < 0.05 versus all other groups) and (**f**) pro-apoptotic (Caspase-3) protein expression (* *p* < 0.05 versus 30 μg/mL groups) using a density meter based on Western blot analysis in hADSCs treated with or without Fe_3_O_4_ nanoparticles. (**g**,**h**) Number of hADSCs nuclei stained with DAPI at 7 days after treating with 9 μg/mL Fe_3_O_4_ nanoparticles and (**i**) its quantification (scale bar indicates 250 μm).

Cytotoxicity, apoptotic activity, and cell proliferation tests were performed to discover whether our Fe_3_O_4_ nanoparticles had appropriate biocompatibility for future biomedical applications. As aforementioned, many studies have reported results using Fe for biomedical applications [30,31,32]. However, Fe ions released from Fe nanoparticles often cause damage and death of animal cells due to excess generation of reactive oxygen species [33,34,35,36]. Therefore, it is important to check the appropriate concentration of Fe-related materials before further biomedical application. As shown in Figure 4a, the relative cell number of hADSCs treated with Fe_3_O_4_ nanoparticles at a concentration of up to 9 μg/mL showed no significant difference from that of hADSCs without any treatment. As shown in Figure 4b, TUNEL staining revealed no statistical difference in TUNEL-positive signals from hADSCs treated with 9 μg/mL of Fe_3_O_4_ nanoparticles compared to the control (hADSCs without nanoparticle treatment) (Figure 4b,c). Apoptotic activity was also tested by analyzing the representative anti-apoptotic (*BCL-2*) and pro-apoptotic (*BAX*) gene expression levels with RT-PCR (Figure 5a) and qRT-PCR analysis (Figure 5c,d). Western blot analysis for cell proliferation markers (PCNA) and apoptosis markers (Caspase-3) was also performed to confirm the results of Figure 5b,e,f at the protein level. Results from RT-PCR, qRT-PCR, and Western blot analysis revealed that there was no significant difference in cell proliferation or apoptotic gene or protein expression between hADSCs treated with 9 μg/mL Fe_3_O_4_ nanoparticles and hADSCs without nanoparticle treatment. For long-term biocompatibility of our synthesized nanoparticles, we checked the proliferative efficacy of hADSCs treated with Fe_3_O_4_ nanoparticles by directly counting the nucleus of hADSCs 7 days after the treatment. As shown in the Figure 5g–i, the number of hADSCs treated with 9 μg/mL Fe_3_O_4_ nanoparticles showed no significant difference compared to that of the control group. Taken together, these results demonstrate that our newly synthesized biocompatible Fe_3_O_4_ nanoparticles might have future biomedical applications.

## 4. Conclusions

Small-sized and uniformly sized magnetite nanoparticles were synthesized by reacting Fe(III) stearate with hydrazine in reverse micelles composed of oleylamine, F127, xylene, and water at a reaction temperature of 90 °C in air atmosphere. In the synthesis, oleylamine acted as a major surfactant to make monodispersed nanoparticles, while F127 made stable reverse micelles in the xylene solution. By controlling experimental conditions including the kind of carboylate ion of the Fe (III) precursor, we could readily succeed in controlling the size of magnetite nanoparticles. For various biomedical applications such as sensing and drug targeting, these uniform and very small magnetic nanoparticles are valuable. We are currently working on the use of these small nanoparticles as a *T_2_* MRI contrast agent. To determine the further application of nanoparticles, cytotoxicity, apoptotic activity, and cell proliferation tests were also performed. Results showed that these newly synthesized Fe_3_O_4_ nanoparticles might be applicable for further biomedical applications. Taken together, our study indicates that the easy and uniform synthetic method for generating biocompatible Fe_3_O_4_ nanoparticles described in the present study might have future biomedical applications.

## Figures and Tables

**Table 1:** Primer sequences used for RT-PCR and qRT-PCR.

Primer Name	Forward Sequence (5′-3′)	Reverse Sequence (5′-3′)
**RT-PCR**	-	-
Human β-actin	GCA CTC TTC CAG CCT TCC TTC C	TCA CCT TCA CCG TTC CAG TTT TT
Human BAX	GTG CAC CAA GGT GCC GGA AC	TCA GCC CAT CTT CTT CCA GA
Human BCL2	TGT GGC TGC ACT TGC TCT AA	CGA TGG CCA TAG ACC CTG TC
**qRT-PCR**	-	-
Human β-actin	CAC CCT GAA GTA CCC CAT CG	TGC CAG ATT TTC TCC ATG TCG
Human BAX	GCA ACT TCA ACT GGG GCC GGG	GAT CCA GCC CAA CAG CCG CTC
Human BCL2	CTT GAC AGA GGA TCA TGC TGT AC	GGA TGC TTT ATT TCA TGA GGC
